# Robot-assisted partial nephrectomy with 3D preoperative surgical planning: video presentation of the florentine experience

**DOI:** 10.1590/S1677-5538.IBJU.2020.1075

**Published:** 2021-06-20

**Authors:** Antonio Andrea Grosso, Fabrizio Di Maida, Riccardo Tellini, Andrea Mari, Simone Sforza, Lorenzo Masieri, Marco Carini, Andrea Minervini

**Affiliations:** 1 University of Florence Unit of Oncologic Minimally-Invasive Urology and Andrology Department of Experimental and Clinical Medicine Florence Italy Department of Experimental and Clinical Medicine, University of Florence - Unit of Oncologic Minimally-Invasive Urology and Andrology, Careggi Hospital, Florence, Italy.

## Abstract

**Purpose:**

Three-dimensional (3D) virtual models have recently gained consideration in the partial nephrectomy (PN) field as useful tools since they may potentially improve preoperative surgical planning and thus contributing to maximizing postoperative outcomes (1-5).

The aim of the present study was to describe our first experience with 3D virtual models as preoperative guidance for robot-assisted PN.

**Materials and methods:**

Data of patients with renal mass amenable to robotic PN were prospectively collected at our Institution from January to April 2020. Using a dedicated web-based platform, abdominal CT-scan images were processed by M3DICS (Turin, Italy) and used to obtain 3D virtual models. 2D CT images and 3D models were separately assessed by two different highly experienced urologists to assess the PADUA score and risk category and to forecast the surgical strategy of the single cases, accordingly.

**Results:**

Overall, 30 patients were included in the study. Median tumor size was 4.3cm (range 1.3-11). Interestingly, 8 (26.4%) cases had their PADUA score downgraded when switching from 2D CT-scan to 3D virtual model assessment and 4 (13.4%) cases had also lowered their PADUA risk category. Moreover, preoperative off-clamp, selective clamping strategy and enucleation resection strategy increased from CT-scan to 3D evaluation.

**Conclusion:**

3D virtual models are promising tools as they showed to offer a reliable assessment of surgical planning. However, the advantages offered by the 3D reconstruction appeared to be more evident as the complexity of the mass raises. These tools may ultimately increase tumor's selection for PN, particularly in highly complex renal masses.

Disclosure of potential conflicts of interest: The authors declare they do not have conflict of interests.

Informed consent: Informed consent was obtained from all individual participants included in the study. All the procedures were in accordance with the ethical standards of the institutional and national research Committee and with the 1964 Helsinki declaration and its later amendments or comparable ethical standards.
